# 

**Published:** 1901-03

**Authors:** 


### CONTENTS OF VOLUME XIX.

*Page*

In Memoriam ... ... ... ... ... ... ... ... ... ... ... ... ... 1

The Uses of Sanatoria for the Treatment of Tuberculosis. By C. Braine-Hartnell, M.R.C.S., L.R.C.P. Lond. ... ... ... 2

Statistics Concerning Consumption and other Preventable Diseases. By W. H. Symons, M.D. Brux., D.P.H. ... ... ... ... ... ... 7

A Retrospect of a Third Series of Fifty Consecutive Intra-Abdominal Operations. By James Swain, M.S., M.D. Lond., F.R.C.S. Eng. 18, 121

Two Cases of Sarcoma of the Intestine, with Secondary Infection in one by a Gas-forming Bacillus. By Theodore Fisher, M.D. Lond., M.R.C.P. Lond. ... ... ... .. ... ... ... ... ... ... 29

On the Question of the Advisability of Ligaturing the Jugular Vein in the Treatment of Sigmoid-Sinus Thrombosis. By J. Lacy Firth, M.S., M.D. Lond., F.R.C.S. Eng. ... ... ... ... ... ... ... 36

Craniotomy, with the Implantation of Egg-shell Membrane, for Jacksonian Epilepsy. By William Jones Greer, F.R.C.S. Irel. 42

Six Cases of Cerebro-Spinal Meningitis. By E. H. Edwards Stack, M.B. Cantab., F.R.C.S. Eng. ... ... ... ... ... ... ... ... ... 44

William Johnstone Fyffe, A.B., M.D. Dub. ... ... ... ... ... ... 97

The Origin of Gastric Ulcer. By W. Gordon, M.A., M.D. Cantab., M.R.C.P. Lond. ... ... ... ... ... .. ... ... ... ... ... 100

Case of Abscess in the Left Lateral Lobe of the Cerebellum, Successfully Evacuated: with Some Remarks on the Method of Operation Employed. By J. Michell Clarke, M.A., M.D. Cantab., F.R.C.P. Lond., and C. A. Morton, F.R.C.S. Eng. ... ... ... 112

A Case of Muscular Atrophy Due to Lead. By J. E. Shaw, M.B. Edin. 117

A Case of Artificial Anus following Herniotomy. By A. W. Prichard M.R.C.S. Eng. ... ... ... ... ... ... ... ... ... ... ... 119

A Case of Presentation of the Axilla at Full Term: Spontaneous Delivery of the Fœtus. By F. Percy Elliott, M.B., C.M. Aberd. 130

X-Ray Photographs as Pictures: with two Diagrams. By William Cotton, M.A., M.D. Edin., D.P.H. ... ... ... ... ... ... ... 131

Alfred Edward Aust Lawrence, M.D., C.M. Aberd. ... ... ... ... 193

CONTENTS.

Page

On Some of the Hemorrhages of Pregnancy. By A. E. Aust Lawrence, M.D. Aberd. ... ... ... ... ... ... ... ... ... 196

The Clinical Significance of Chronic Hoarseness and Loss of Voice. By P. Watson Williams, M.D. Lond. ... ... ... ... ... .. 203

A Case of Paralysis of the Right Vocal Cord, due to Thoracic Aneurysm. By Barclay J. Baron, M.B. Edin. ... ... ... ... 208

The Tuberculin Test in Cattle. By G. S. Pollard, L.R.C.P. Edin., M.R.C.S. Eng. ... ... ... ... ... ... ... ... ... ... ... 210

Some Cases of Displacement of Abdominal Viscera. By Theodore Fisher, M.D. Lond., M.R.C.P. Lond. ... ... ... ... ... ... 217

Notes on Two Hundred and Twelve Consecutive Operations in Four Thousand Six Hundred and Ten Cases among the Medical Patients, Bristol Royal Infirmary. By E. H. Edwards Stack, M.B. Cantab., F.R.C.S. Eng. ... ... ... ... ... ... ... 225, 326

The Progress in the Diagnosis and Treatment of Affections of the Throat, Nose and Ear during the Past Twenty Years. By Barclay J. Baron, M.B. Edin. ... ... ... ... ... ... ... ... ... 289

Cystoscopy and Ureteral Catheterization. Observations on Twenty Examinations for Obscure Urinary and Abdominal Affections. By Thos. Carwardine, M.S. Lond., F.R.C.S. Eng. ... ... ... ... ... 303

Some Surgical Aspects of Meckel's Diverticulum. By William Sheen, M.S., M.D. Lond., F.R.C.S. Eng. ... ... ... ... ... ... 310

The Quiet Production of Anæsthesia. A Clinical Lecture. By John Freeman, F.R.C.S. Edin. ... ... ... ... ... ... ... ... ... 321

The Fourteenth International Congress of Medicine ... 286, 382

Progress of the Medical Sciences ... ... ... 51, 136, 234, 334

Reviews of Books ... ... ... ... ... ... ... 65, 151, 245, 346

Editorial Notes ... ... ... ... ... ... ... ... 76, 167, 274, 363

Notes on Preparations for the Sick ... ... ... 79, 171, 280, 370

Obituary ... ... ... ... ... ... ... ... 82, 97, 173, 193, 366, 381

Local Medical Notes ... ... ... ... ... ... 83, 187, 286, 379

Meetings of Societies ... ... ... ... ... ... ... 85, 173, 375

The Library of the Bristol Medico-Chirurgical Society ... ... ... ... ... ... ... ... ... 91, 183, 282, 373

